# Machine Learning-Assisted Burst Femtosecond Laser Polishing of Invar Alloy: Process Optimization and Performance Enhancement

**DOI:** 10.3390/nano16060383

**Published:** 2026-03-23

**Authors:** Jiawei Lin, Donghan Li, Jinlin Luo, Kai Li, Xianshi Jia, Cong Wang, Xin Li, Ke Sun, Ji’an Duan

**Affiliations:** 1 State Key Laboratory of Precision Manufacturing for Extreme Service Performance, College of Mechanical and Electrical Engineering, Central South University, Changsha 410083, China; 243712133@csu.edu.cn (J.L.); 253711072@csu.edu.cn (D.L.); 233712115@csu.edu.cn (J.L.); likai01@csu.edu.cn (K.L.); wangcong@csu.edu.cn (C.W.); duanjian@csu.edu.cn (J.D.); 2 State Key Laboratory of Pulsed Power Laser Technology, College of Electronic Engineering, National University of Defense Technology, Hefei 230000, China; lixinkiller@nudt.edu.cn (X.L.); sunke08@nudt.edu.cn (K.S.); 3 Anhui Laboratory of Advanced Laser Technology, Hefei 230037, China; 4 Nanhu Laser Laboratory, Changsha 410073, China

**Keywords:** femtosecond laser, laser polishing, invar alloy

## Abstract

As a key low-expansion material for high-end equipment such as aerospace and precision instruments, the surface quality of Invar alloy directly determines the operational performance of devices. To fill the research gap in the multi-parameter synergy and mechanism of Invar alloy laser polishing, this study performs polishing experiments on Invar alloy using a burst-mode femtosecond laser, with a repetition rate of 1 MHz and four sub-pulses per burst. The results indicate that energy density plays a dominant role in the polishing effect: with the increase in energy density, the surface roughness first decreases and then increases. A stable molten pool is formed under medium energy density (0.47–0.64 J/cm^2^), and under the optimal parameter conditions, the surface roughness is reduced to 394 ± 50 nm, representing a 52% reduction compared to the original surface (821 nm). Scanning speed and scanning pitch affect the polishing effect by synergistically regulating energy input: increasing scanning speed under high energy density can inhibit the rise in roughness, while a small scanning pitch can lower the threshold of optimal energy density. Amplitude spectrum analysis reveals that the medium-scale surface undulations are significantly improved after polishing. A four-layer Fully Connected Neural Network (FCNN) model is established to achieve high-precision prediction of polishing effects with a coefficient of determination R^2^ = 0.92, which enables rapid prediction of unknown polishing parameter combinations and provides a new solution path for the optimization of polishing effects. This study clarifies the interaction mechanism between a burst-mode laser and Invar alloy, proposes an efficient ultra-precision polishing method for Invar alloy, and lays a theoretical foundation for its application in the field of high-end manufacturing.

## 1. Introduction

In the fields of modern precision manufacturing and high-end equipment, Invar alloy, a low-expansion precision alloy based on iron and nickel, has become a key material for temperature-sensitive devices due to its extremely low coefficient of thermal expansion at room temperature [1,2]. It has found irreplaceable applications in high-tech fields such as aerospace [3], precision instruments [4], high-end electronic devices [5], and metrological equipment [6]. With the technological upgrading in third-generation semiconductors, deep space exploration, and other fields, higher requirements have been put forward for the precision machining of Invar alloy components, including surface roughness and dimensional accuracy. Thus, a polishing step is typically required after multiple prior processing procedures to improve the surface quality of workpieces. For example, when used as a pre-processing procedure, polishing is also of great significance for the welding of heterogeneous materials. When conducting heterogeneous welding between metal materials such as Invar alloy and glass materials, metal materials with high surface quality facilitate the achievement of heterogeneous joints with excellent bonding strength [7,8]. Therefore, as a critical process in the precision manufacturing of Invar alloy, surface polishing directly affects the operational performance of Invar alloy components.

Existing polishing technologies generally have insurmountable drawbacks. For example, mechanical polishing removes rough surface parts through mechanical cutting and plastic deformation, which is inefficient and difficult to process parts with complex shapes [9]; chemical polishing achieves polishing by directly dissolving surface protrusions through chemical reactions, but the polishing solution has a short service life and the processing method is not environmentally friendly [10]; electrolytic polishing involves placing the workpiece in a specific electrolyte, where surface protrusions are preferentially dissolved through electrolysis, but it requires many pre-treatment steps and has a complex process [11]; and ultrasonic polishing uses the cavitation effect of ultrasonic waves to make abrasive particles in the polishing solution perform micro-impacts on the metal surface, removing fine scratches and protrusions, but it has high requirements for equipment and process parameters and a relatively narrow scope of application [12]. Therefore, traditional polishing methods currently have various shortcomings, and new polishing methods are needed to meet the precision machining requirements of Invar alloy.

The continuous development of laser technology has shown its potential in many processing fields [13,14,15,16,17,18,19,20,21,22,23], including laser drilling [24,25,26], surface treatment [27], laser cutting [28,29], and laser additive manufacturing [30,31]. Laser polishing technology provides a new solution for the precision machining of materials [32,33]. Compared with traditional polishing technologies, laser polishing technology has significant advantages. Firstly, laser polishing is a non-contact processing method, which is more flexible and easier to process brittle materials. Secondly, laser polishing has high efficiency, especially when processing large-area or complex-shaped workpieces, which can significantly improve production efficiency and is suitable for automated production. Thirdly, laser polishing has many adjustable parameters, which can meet different processing needs and environments [34]. In recent years, remarkable progress has been made in the research of this technology on materials such as stainless steel, titanium alloy, and superalloy.

According to existing research, the laser polishing process is dominated by melting, especially for continuous wave pulse lasers or long pulse lasers. The mechanism of laser polishing is as follows. After the laser acts on the sample, it injects heat, causing the sample material to heat up and melt to form a molten pool. The molten pool is fluid, so its surface gradient is weakened compared to the original surface. After the laser action ends, the molten pool solidifies again, forming a relatively smooth surface and achieving the polishing effect [35,36]. Changing polishing parameters, including laser parameters (e.g., repetition rate, energy, spot size) and process parameters (e.g., scanning speed, scanning pitch, number of scans), can all affect the final polishing effect.

Relevant studies on laser polishing have been conducted. Chen et al. polished silicon carbide materials using a femtosecond laser and studied the processing parameters of an obliquely incident laser, including the effects of incident angle, overlap rate, energy, and scanning times on the polishing effect. The incident angle was the main factor. It was found that compared with a vertically incident laser, large incident angle laser polishing can significantly reduce surface roughness. When the laser incident angle is set to 80 degrees, the pulse energy is 85 μJ, the X-direction overlap rate is 90%, the Y-direction overlap rate is 95%, the laser repetition rate is 35 kHz, and the scanning times is six, the optimal polished roughness of silicon carbide ceramics is 0.74 μm [37]. Jaritngam et al. studied nanosecond laser polishing of titanium alloy (Ti6Al4V), focusing on the effects of laser power, laser repetition rate, and scanning speed on the polishing effect. They proposed that higher laser power or slower scanning speed would lead to a thicker recast layer, higher oxidation degree, a larger heat-affected zone, and more microcracks [38]. Huang et al. studied a processing method for polishing zirconium-based metallic glass using a nanosecond laser, and systematically investigated the effects of parameters such as laser power, scanning speed, and overlap rate on the surface microstructure, surface roughness, and surface hardness of metallic glass during laser polishing. The results show that nanosecond laser polishing can reduce the surface roughness of metallic glass by at least 72.9%, and the surface hardness and mechanical properties are improved [39].

In addition to the research on the effects of laser parameters on polishing results, some studies have begun to explore more complex laser polishing process steps or attempt algorithmic analysis and prediction of polishing effects. Loubère et al. studied the effects of femtosecond laser parameters on the surface roughness and ablation thickness of pure copper, and further explored a two-step processing strategy consisting of rough polishing and fine polishing. They studied the changes in surface profile after each polishing step and achieved a reduction in initial surface roughness from 15 μm to less than 400 nm [40]. Pong-Ryol et al. used two lasers: an ultraviolet nanosecond pulse laser for polishing and a continuous wave laser for laser spot control. By detecting and adjusting the focal offset to control the laser spot size on the material surface, the laser flux of the ultraviolet nanosecond pulse laser on the surface was kept constant, further improving the laser micro-polishing efficiency. The roughness of the inclined surface was reduced from the original value to 85.70 nm, a decrease of 56.4%, while that of the curved surface was reduced to 82.30 nm, a decrease of 57.3% [41]. Kumar et al. studied the polishing of stainless steel with a line-focused beam, investigated the surface shallow melting and over-melting phenomena under different energy densities, and adopted an experimental design based on response surface methodology for multi-objective optimization, thereby realizing the prediction of polished roughness within the parameter space. They also studied the mechanical properties of the polished stainless steel surface [42].

Meanwhile, relevant studies have also explored laser polishing in burst mode [43,44]. For femtosecond lasers, due to their ultra-short pulse duration, materials are typically ablated via the high energy input delivered by a single short pulse [45]. In the context of femtosecond laser polishing, related works have reported that polishing can be achieved through non-thermal effects, namely direct ablation. For example, Taylor et al. proposed a femtosecond laser polishing method for germanium and systematically investigated the influence of laser parameters on surface morphology, roughness, and material removal mechanism. Their results indicated that, under low repetition rate and single pulse mode, the femtosecond laser realizes nearly non-thermal ablation polishing of germanium, with no obvious remelting layer or heat-affected zone [46]. However, when femtosecond lasers operate at high repetition rates or further adopt burst mode, the polishing mechanism transitions to a melting-dominated regime similar to that observed with continuous-wave laser pulses [47]. Burst mode utilizes ultra-high repetition rates to enable a controlled, thermally driven polishing mechanism. The ultra-short time interval between adjacent sub-pulses allows efficient residual heat reuse: thermal energy deposited by preceding pulses preheats the material surface, lowering the melting threshold and facilitating the formation of a shallow and stable molten pool. Because of the shallow molten layer, no significant thermal deformation occurs upon cooling. The combination of ultra-short intra-burst pulse intervals and the intrinsic characteristics of femtosecond pulses restrict heat penetration into the bulk material. In addition, burst mode introduces extra adjustable parameters related to sub-pulses, such as the number of sub-pulses per burst, the intra-burst repetition rate, and the sub-pulse interval. These parameters allow more refined control over the energy accumulation effect and molten pool formation, thus offering greater potential for advanced laser polishing.

However, there is currently a lack of multi-parameter and multidimensional research on burst-mode laser polishing of Invar alloy, and the synergistic effects of key processing parameters as well as the underlying polishing mechanisms remain unexplored. Therefore, this paper explored the laser polishing of Invar alloy by using a burst-mode femtosecond laser. The effects of three different parameters (energy density, scanning speed, scanning pitch) on polishing roughness were studied. The polishing mechanism was analyzed by combining Scanning Electron Microscopy (SEM) images and Energy Dispersive Spectroscopy (EDS) analysis, and further research was conducted using amplitude spectrum and deep learning methods.

## 2. Materials and Methods

### 2.1. Equipment and Materials

**Figure 1:**
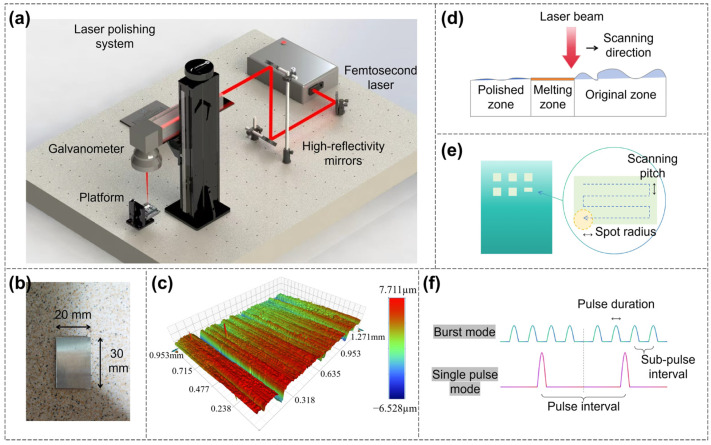
(**a**) Laser polishing system. (**b**) Invar alloy sample. (**c**) Original surface of the Invar alloy sample. (**d**) Schematic diagram of laser polishing. (**e**) Laser scanning path. (**f**) Schematic diagram of burst mode.

The laser polishing system, schematically illustrated in Figure 1a, primarily consisted of a femtosecond laser source (HR-Platform-0203, Wuhan Huaray Precision Laser Co., Ltd., Wuhan, China), three high-reflectivity mirrors, a galvanometer scanning system, and a precision sample stage. The femtosecond laser beam was directed into the galvanometer scanning system via high-reflectivity mirrors, which regulated the scanning path on the sample surface. Meanwhile, the sample was firmly fixed on the precision stage to maintain positional stability throughout the polishing process.

For the fixed laser parameters, the femtosecond laser beam is a Gaussian beam, the wavelength of the femtosecond laser is 1035 nm, the pulse duration is 200 fs, the repetition rate is 1 MHz, the number of sub-pulses in burst mode is four (shown in Figure 1f), the sub-pulse repetition rate within the burst is 22 MHz, and the diameter of the femtosecond laser spot is 30 μm.

The adjustable process parameters and their corresponding ranges selected are as follows: single pulse energy density ranging from 0.35 to 0.85 J/cm^2^, scanning speed ranging from 20 to 80 mm/s, and scanning pitch fixed at two levels of 0.5 μm and 1 μm. Energy density refers to fluence per pulse, defined as the energy of a single femtosecond laser pulse divided by the area of the laser spot. More specifically, F = E/A, where F is the energy density, E is the energy of a single femtosecond laser pulse, and A is the area of the laser spot. In burst mode, the energy of a single pulse refers to the total energy of the undivided pulse rather than the energy of each sub-pulse.

The Invar alloy samples (shown in Figure 1b) used in the experiment have a size of 30 mm × 20 mm × 2 mm. As shown in Figure 1c, the original surface of the Invar alloy sample used in this experiment had scratches of different sizes in a single direction. The roughness of the original surface was 821 nm (average of 10 measurement results), which is used as a benchmark for comparison with subsequent polished results.

### 2.2. Methods

Prior to laser polishing, the samples were ultrasonically cleaned to remove surface contaminants. The samples were then polished through laser scanning (shown in Figure 1d). During polishing, the laser focus was maintained on the upper surface of the sample, corresponding to a fixed defocus of 0. A zigzag scanning path was used, (shown in Figure 1e), with the path direction perpendicular to the original scratches. The laser only performed a single scan for polishing, and the size of polished area was set to 1.5 mm × 1.5 mm. For each parameter combination, four repeated trials were conducted, and the average Sa value was reported to improve data reliability.

### 2.3. Characterization

The surface morphology of the samples was initially observed using an optical microscope (NM710, Ningbo Yongxin Optics Co., Ltd., Ningbo, China).

Three-dimensional surface topographies before and after polishing were measured using a profilometer (ContourGT, Bruker Nano Surfaces, Bruker Corporation, Billerica, MA, USA). The profile data were processed using Vision 64 software to obtain the corresponding surface roughness Sa as the main data. Before calculating the roughness, tilt correction was performed using the software’s built-in function. The size of the profilometer measurement area was set to 1.271 mm × 0.953 mm, which was slightly smaller than the size of polished area, and the measurement area was located at the center of the polished area to ensure the validity of the measurement data.

Scanning Electron Microscopy (SEM, TM4000 PLUS, Hitachi High-Tech Corporation, Tokyo, Japan) was employed to further observe the surface morphology of the samples before and after polishing. Energy Dispersive Spectroscopy (EDS, Ultim Max 40, Oxford Instruments NanoAnalysis, Oxford Instruments plc, Abingdon, Oxfordshire, UK) was used to analyze the elemental composition (Fe, Ni, O) and distribution on the surface.

## 3. Results and Discussion

### 3.1. Effect of Energy Density on Polishing Performance

**Figure 2:**
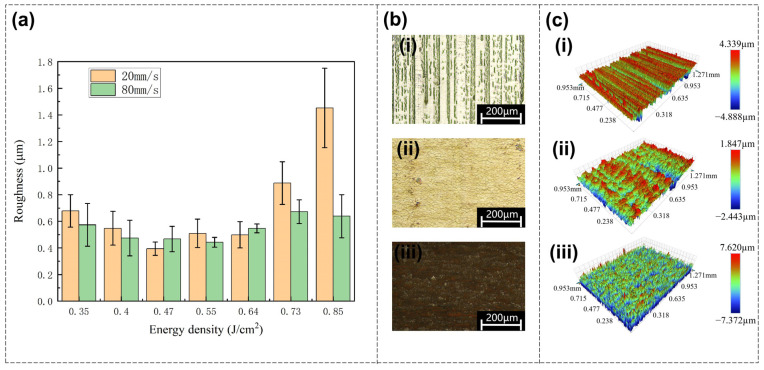
(**a**) Relationship between polished surface roughness and increasing energy density, showing an overall trend of first decreasing and then increasing. The scanning pitch is fixed at 1 μm, and the scanning speeds are 20 mm/s and 80 mm/s, respectively. (**b**) Optical images of the polished surface at different energy densities when the scanning speed is 20 mm/s: (**i**) 0.35 J/cm^2^; (**ii**) 0.55 J/cm^2^; (**iii**) 0.85 J/cm^2^. (**c**) Three-dimensional images of the polished surface at different energy densities when the scanning speed is 20 mm/s, corresponding to (**b**): (**i**) 0.35 J/cm^2^; (**ii**) 0.55 J/cm^2^; (**iii**) 0.85 J/cm^2^.

During the femtosecond laser polishing of Invar alloy samples, the laser energy density has the most significant impact on the final roughness. As shown in Figure 2a, the laser scanning pitch is fixed at 1 μm, and the data series corresponds to scanning speeds of 20 mm/s and 80 mm/s, respectively. At these fixed parameters, the surface roughness of the Invar alloy sample after polishing first decreases and then increases with the increase in energy density.

At a scanning speed of 20 mm/s, the polished surface roughness decreases as the energy density increases from 0.35 J/cm^2^ onwards. At an energy density of 0.47 J/cm^2^, the surface roughness reaches a minimum value of 394 nm, followed by a plateau period where the surface roughness remains relatively stable with slight fluctuations. After the energy density reaches 0.64 J/cm^2^ and above, the surface roughness begins to increase significantly. Finally, when the energy density reaches the maximum value of 0.85 J/cm^2^, the surface roughness rises to 1453 nm. Therefore, according to the change trend of surface roughness, the optimal polishing effect of Invar alloy is achieved under medium energy density.

The optical images of the polished surface under different energy densities at a scanning speed of 20 mm/s are shown in Figure 2b, from which one can initially observe the differences in the surfaces corresponding to each energy density. The corresponding three-dimensional morphologies of the surfaces are shown in Figure 2c.

**Figure 3:**
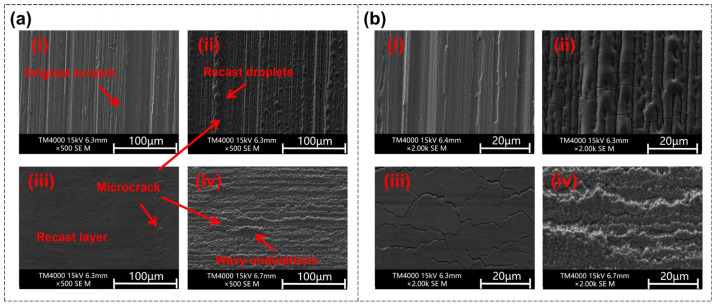
(**a**) SEM images of the original surface and the polished surface under different energy densities: (**i**) Original surface with vertical shallow scratches; (**ii**) low energy density (0.35 J/cm^2^) with recast droplets and slight polishing effect; (**iii**) medium energy density (0.55 J/cm^2^) with recasting after the formation of an overall molten pool and optimal polishing effect; (**iv**) high energy density (0.85 J/cm^2^) with wavy undulations and increased roughness exceeding the original surface. (**b**) SEM images of the original surface and the polished surface under different energy densities, enlarged version of the corresponding image in (**a**).

In order to further observe the surface morphology and analyze the polishing mechanism, SEM was used to capture images of the original surface and polished surfaces at different energy densities, with a fixed scanning pitch of 1 μm and a scanning speed of 20 mm/s. As shown in Figure 3a(i), the original surface has scratches of different sizes in a single direction, which are surface defects that need to be eliminated by polishing. The polished surfaces exhibit distinctly different morphological features depending on the applied energy density.

Polishing at a low energy density (0.35 J/cm^2^) yields a surface featuring recast droplets, as illustrated in Figure 3a(ii). At this energy density, the molten Invar alloy during polishing is insufficient to form a full-coverage molten pool, leading to the formation of regionally distributed droplet-like structures that solidify upon cooling. In this case, the polishing effect is relatively slight, thus the vertical scratches on the surface, especially the larger ones, are difficult to remove under this low energy density, so the roughness is not significantly reduced.

As the energy density increases to a medium level of 0.55 J/cm^2^, the change in the polished surface compared to the original surface is remarkable (shown in Figure 3a(iii)). At this energy density, a complete molten pool covering the entire action area is formed during the polishing process. Due to the flow of the molten pool from high to low, the surface is flattened, and this flatness is retained after solidification. The shallow scratches on the original surface are eliminated, although there are still a few traces of deeper scratches. At this energy density, the roughness of the polished surface is reduced to the greatest extent.

Further increasing the energy density to 0.85 J/cm^2^, as shown in Figure 3a(iv), the defects and other characteristics of the original surface are completely invisible on the polished surface, but new defects appear. At higher energy density, excessive thermal energy input induces molten pool instability, resulting in violent sputtering and intense fluctuations. Despite the complete elimination of vertical scratches, new wavy undulations form during solidification, leading to surface roughness exceeding that of the original surface. Therefore, under high energy density, a positive polishing effect is not actually achieved—that is, the surface quality deteriorates.

In addition, microcracks can be observed on the polished surface under any energy density, which originate from the cooling shrinkage stress of the surface recast layer during the process of cooling from high temperature to room temperature. However, the distribution of microcracks is relatively uniform under medium energy density.

From the above analysis, in the process of polishing Invar alloy using a burst-mode laser, the flattening and recasting of the molten pool are the dominant mechanisms of polishing, meaning hot polishing is the main mechanism, and therefore energy density becomes the key factor that dominates the polishing effect. The polished surface morphology changes significantly with increasing energy density. There are two distinct thresholds for energy density, and between these two thresholds, at a medium energy density, a relatively optimal polishing effect is achieved.

**Figure 4:**
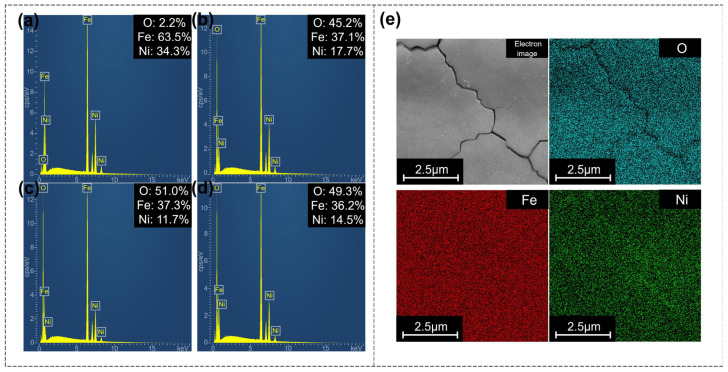
(**a**) Element content of the original surface. (**b**) Element content at low energy density (0.35 J/cm^2^). (**c**) Element content at medium energy density (0.55 J/cm^2^). (**d**) Element content at high energy density (0.85 J/cm^2^). (**e**) Element distribution on the polished surface at medium energy density, including oxygen, iron, and nickel elements.

Energy Dispersive Spectroscopy (EDS) was utilized to analyze the elemental composition (Fe, Ni, O) and distribution on both the original and polished surfaces. As shown in Figure 4, according to the element content results, the oxygen content on the polished surface increases significantly even at the lowest energy density, while the iron content decreases slightly and the nickel content decreases significantly. This indicates that obvious oxidation occurs during the polishing process, and the elements in the high-temperature molten pool are more likely to react with oxygen in the air to cause oxidation. From the element distribution results, the oxygen content in the microcracks is slightly lower than that in the surrounding areas, likely because the oxides are mainly distributed in the thin recast layer on the surface and have not entered the interior of the material.

### 3.2. Effect of Scanning Speed and Scanning Pitch on Polishing Performance

Although energy density is the dominant factor governing polishing performance, the synergistic influences of scanning speed and scanning pitch should also be considered.

**Figure 5:**
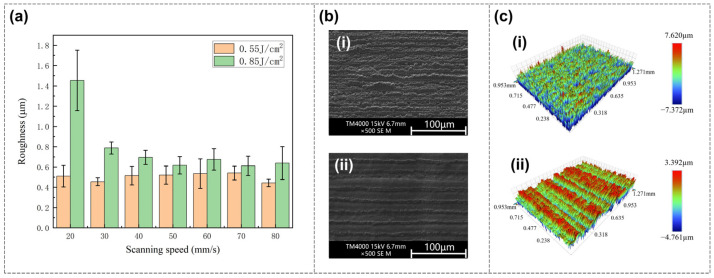
(**a**) Relationship between polished surface roughness and increasing scanning speed. (**b**) SEM images under high energy density (0.85 J/cm^2^): (**i**) Lowest scanning speed (20 mm/s); (**ii**) highest scanning speed (80 mm/s). (**c**) Three-dimensional images corresponding to (**b**): (**i**) Lowest scanning speed; (**ii**) highest scanning speed.

Figure 5a depicts the surface roughness after polishing at different scanning speeds. According to the polished roughness results, the change in scanning speed has no obvious effect on the roughness under low or medium energy density, while the surface roughness decreases with the increase in scanning speed under high energy density. This indicates that there is a synergistic effect between various parameters on the polishing effect. For low or medium energy density, although the laser scanning speed will affect the heat input, the energy density plays a major role, so the scanning speed has little effect on the final roughness. For high energy density, the over-melting state has been entered. A lower scanning speed corresponds to more energy input, which will lead to greater instability of the molten pool, thus increasing the roughness.

Figure 5b shows the SEM images under high energy density (0.85 J/cm^2^), where (i) is the lowest scanning speed (20 mm/s) and (ii) is the highest scanning speed (80 mm/s). Due to the high energy density, both have over-melting phenomena, but there are differences. The energy input at the lowest scanning speed is larger, and wavy undulations appear in the entire area with a higher degree of undulation. At the highest scanning speed, the energy input is smaller, and a unique morphology of mixed over-melting areas and shallow melting areas can be seen. These two types of areas alternately appear in the direction perpendicular to the scanning path, and the undulation of the over-melting areas is also smaller, so the increase in roughness is not obvious. Figure 5c shows the corresponding three-dimensional images.

**Figure 6:**
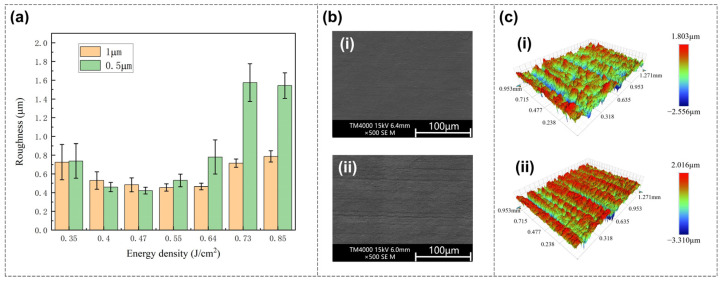
(**a**) Effect of different scanning pitches on polished roughness. A small scanning pitch increases energy input, thus achieving the optimal polishing effect at a lower energy density, while a large scanning pitch requires a higher energy density to achieve the optimal polishing effect. (**b**) SEM images of the polished surface with optimal parameters under each scanning pitch: (**i**) 1 μm; (**ii**) 0.5 μm. (**c**) Three-dimensional images of the polished surface with optimal parameters under each scanning pitch: (**i**) 1 μm; (**ii**) 0.5 μm.

The influence of scanning pitch on polishing performance was further investigated. As shown in Figure 6a, the relationship between roughness and energy density under different scanning pitches (1 μm and 0.5 μm) is presented, with the scanning speed set to 30 mm/s. For both pitches, roughness decreases initially and then increases with increasing energy density; however, the energy density yielding the minimum roughness shifts with scanning pitch. Specifically, the minimum occurs at 0.47 J/cm^2^ for the smaller pitch (0.5 μm) and at 0.55 J/cm^2^ for the larger pitch (1 μm).

This phenomenon can be attributed to the effect of scanning pitch on effective energy input: a smaller scanning pitch results in repeated energy deposition in adjacent regions, leading to higher cumulative energy input and thus a lower energy density threshold for optimal polishing. In addition, the over-melting effect is more obvious under a small scanning pitch and high energy density. In conclusion, the scanning pitch can be considered to indirectly affect the energy density.

Based on the analysis of the three key parameters mentioned above, energy density exerts the most significant influence on the laser polishing effect, while scanning pitch and scanning speed also contribute. Additionally, the synergistic effects of these three parameters on polishing performance cannot be overlooked.

### 3.3. Comparison of Single Pulse Mode Polishing

To further clarify the melting-dominated mechanism of the burst-mode femtosecond laser polishing and study the difference between femtosecond single pulse mode and burst-mode interaction mechanisms, a single pulse femtosecond laser was used to polish Invar alloy under the same basic laser parameters, with a fixed scanning pitch of 1 μm, a fixed scanning speed of 30 mm/s, and energy density as a variable. Comparisons between the differences in polishing morphology and interaction mechanism of the two modes based on experimental results are as follows.

**Figure 7:**
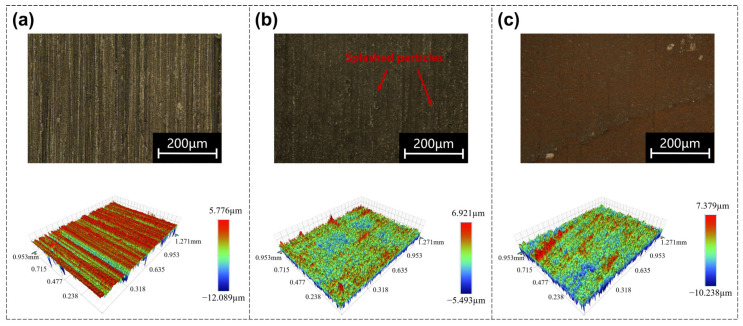
Polishing results in single pulse mode. (**a**) 0.12 J/cm^2^, only a certain thermal effect is generated in the processing area; (**b**) 0.15 J/cm^2^, the surface begins to be ablated, and the splashed particles around the area are a typical phenomenon of ablation; (**c**) 0.55 J/cm^2^, significant increase in surface roughness under high energy density.

As shown in Figure 7, in single pulse mode, the energy density required to induce changes in the surface structure of the material is relatively low. At low energy densities (<0.15 J/cm^2^), only a certain thermal effect is generated in the processing area, and there are no obvious signs of material melting or ablation. When the energy density increases to a certain degree (≥0.15 J/cm^2^), obvious ablation phenomena are observed on the polished surface, accompanied by splashing particles around the polishing area, which are typical features of material evaporation and removal. For roughness, there is no significant decrease at low energy densities: at the beginning of entering the high-energy density ablation state, the roughness slightly decreases (675 nm at 0.15 J/cm^2^); as the energy density further increases, reaching 0.55 J/cm^2^, it is in a stable polishing effect state in the corresponding burst mode. However, in the single pulse mode, the energy density is too high and leads to severe ablation. The single pulse laser causes excessive ablation and disordered redeposition of the material surface, resulting in a significant increase in roughness. As a comparison, in the corresponding burst mode (as shown in Figure 2b(ii)), a complete and smoother recast layer is formed.

Based on the above phenomenon, the differences in polishing between single pulse mode and burst mode are compared. In the single pulse mode, the ultra-high peak intensity of a single femtosecond pulse directly induces ablation on the Invar alloy surface. The peak intensity exceeds the ablation threshold of Invar alloy, leading to direct evaporation and removal of surface materials. In the burst mode adopted in this study, the laser pulse is divided into four sub-pulses with equal energy distribution. The energy of a single sub-pulse is lower than the ablation threshold of Invar alloy, so it cannot directly cause material ablation. Instead, the energy of each sub-pulse is converted into internal energy of the material, causing localized heating of the surface. The intra-burst sub-pulse interval is shorter than the thermal relaxation time of Invar alloy, resulting in significant residual heat accumulation between adjacent sub-pulses. This thermal accumulation effect causes the surface temperature of the material to rise rapidly until it exceeds the melting point, forming a shallow and stable molten pool. The polishing effect is ultimately achieved through the flattening of the molten pool during the melting-solidification process.

### 3.4. Further Analysis and Performance Prediction

#### 3.4.1. Amplitude Spectrum Analysis

**Figure 8:**
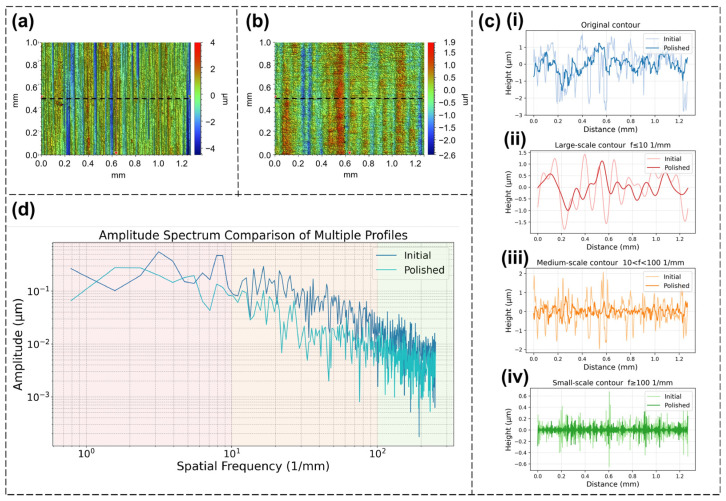
(**a**) Original surface: the dotted line position is the x-direction profile extraction position; (**b**) polished surface with optimal parameters: the dotted line position is the x-direction profile extraction position; (**c**) (**i**) the line profile at the dotted line position in the original surface and polished surface; (**ii**–**iv**) the surface undulations of different scales obtained by decomposing the original line profile; (**ii**) large scale, (**iii**) medium scale, (**iv**) small scale; (**d**) amplitude spectrum diagram obtained by processing the profile.

Although the areal roughness used in the previous analysis can intuitively reflect the surface quality, some details are lost. Here, through amplitude spectrum analysis, the undulations of the Invar alloy sample surface at different scales can be studied to supplement some details. As shown in Figure 8a, a line profile in one direction is extracted from the three-dimensional image of the original surface or the polished surface using software. To analyze the characteristics more clearly, the selected direction is set to be perpendicular to the original scratches of the sample, that is, the x-direction. The extracted line profile of the original surface is shown in Figure 8c(i).

The line profile is converted into an amplitude spectrum diagram through fast Fourier transform (FFT), as shown in Figure 8d. The figure shows the amplitude spectra of the original surface and the polished surface with optimal parameters. Based on the range of frequencies, the amplitude spectra are divided into large-scale (f ≤ 10 mm^−1^), medium-scale (10 mm^−1^ < f < 100 mm^−1^), and small-scale parts (f ≥ 100 mm^−1^). According to the amplitude spectra, the surface undulations at medium scale (orange part) are significantly reduced, which mainly contributes to the reduction in roughness, while the reduction in surface undulations at large scale (red part) and small scale (green part) is relatively small. In addition, for each frequency interval, after extracting the amplitude signal, the amplitude can be converted back from the frequency domain to the spatial domain through inverse fast Fourier transform (iFFT), resulting in decomposed surface line profiles at different scales for further observation (Figure 8c(ii–iv)).

Combined with the analysis, for large-scale surface undulations the gradient of surface height is low, so the fluidity of the molten pool after formation is insufficient to compensate for the surface undulations with a low gradient, resulting in insufficient weakening of large-scale surface undulations. Small-scale surface undulations are derived from the microstructures on the surface, so they cannot be well weakened by polishing.

Among these, small-scale undulations have a relatively minor impact on roughness, while large-scale undulations have a significant impact. Therefore, in order to further improve the polishing effect, other process steps may need to be taken to remove large-scale undulations, such as first etching the material or attempting multiple scanning polishing.

#### 3.4.2. Deep Learning Prediction

For laser polishing processes, the large number of process parameters and their combinations necessitates the use of algorithms to predict the polishing outcomes of untested parameter sets, thereby facilitating the identification of optimal parameters and enhancing research efficiency. These algorithms include classic polynomial regression, response surface methodology, etc. Through deep learning methods, known data can also be used to predict unknown data or predict the parameters required to achieve the optimal effect.

**Figure 9:**
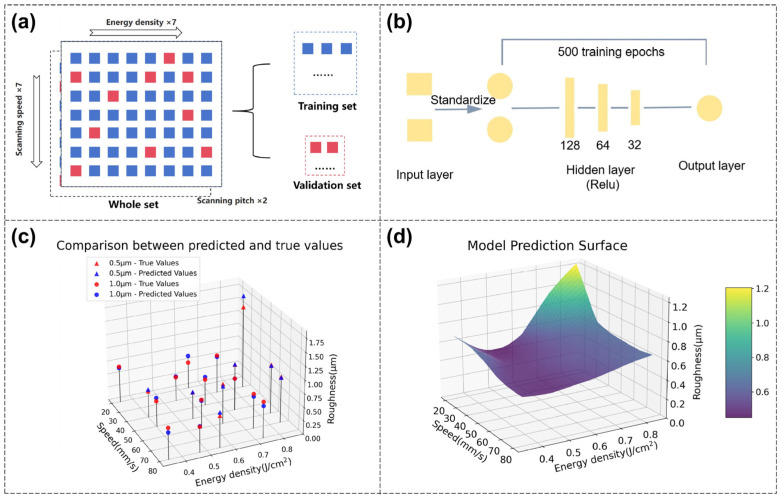
Establishing a model between polishing parameters and polished roughness through deep learning. (**a**) Establishment and division of the dataset: blue represents training set data (accounting for 80%), red represents test set data (accounting for 20%), and the goal is to establish a model through the polishing parameters and polished roughness in the training set. (**b**) Schematic diagram of model architecture. (**c**) True values and predicted values on test set, with the coefficient of determination R^2^ reaching 0.92. (**d**) The model’s prediction of the roughness of the parameter space (the scanning pitch is 1 µm), which can predict the polished roughness corresponding to unused laser parameter combinations.

In this section, a model between polishing parameters and polished roughness is established through deep learning. The goal is to train the model through the training set and verify it through the test set. The obtained model can predict the polished roughness corresponding to unused laser parameter combinations. The total number of experimental data points used for the model is 98, which is derived from seven energy density levels (0.35–0.85 J/cm^2^), seven scanning speed levels (20–80 mm/s), and two scanning pitch levels (0.5 μm, 1 μm). The data are divided into training set data and test set data in the ratio of 80% and 20% (shown in Figure 9a).

Due to the small size of the dataset, a simple and mature Fully Connected Neural Network (FCNN) model was adopted to avoid overfitting and achieve faster processing speed. The model architecture is set to FCNN with four layers and ReLU as the activation function (shown in Figure 9b). In the data preprocessing stage, StandardScaler is used to standardize the input features. The model training uses mean squared error (MSE) as the loss function and stochastic gradient descent (SGD) as the optimizer, with a total of 500 training epochs.

Validation results show close agreement between predicted and measured roughness, yielding an R^2^ of 0.92 (Figure 9c), which supports the feasibility of predicting polished roughness from laser parameters. The prediction of the roughness of the entire parameter space is presented in Figure 9d. The original laser processing data are discrete, meaning there are intervals between parameter values. However, the deep learning model trained on these discrete data can predict the polished roughness for continuous parameter combinations within the original parameter space. Therefore, for unused laser parameter combinations, their polished roughness can be predicted, thereby finding suitable polishing parameters more efficiently. For application in different burst-mode configurations or scanning pitch values, additional experimental data need to be supplemented for model retraining, which will be further explored in future research.

## 4. Conclusions

This study investigated the effect of femtosecond laser polishing of Invar alloy in burst mode. The effects of three different parameters on the polishing effect and the synergistic effect between each parameter were studied by combining the three-dimensional morphology and roughness measured by the profilometer, SEM images, and EDS analysis. Further analysis was conducted using amplitude spectrum analysis and deep learning methods. The main conclusions are as follows:

(1) The energy density has the most significant impact on polishing, and the polishing effect is optimal at medium energy density. The optimal process parameters for femtosecond laser polishing of Invar alloy in burst mode are an energy density of 0.47 J/cm^2^, scanning speed of 20 mm/s, and scanning pitch of 1 μm. With these parameters, the surface roughness Sa decreases from 821 nm to 394 ± 50 nm, a decrease of 52%;
(2) The polishing in burst mode is dominated by thermal polishing, so the scanning speed and scanning pitch affect the energy input and synergistically affect the polishing effect with energy density, providing more parameter control space;
(3) The FCNN deep learning model can accurately establish the mapping relationship between polishing parameters and roughness (R^2^ = 0.92) and predict the achievable roughness of unknown parameter combinations, thereby significantly enhancing the efficiency of parameter selection and paving the way for further optimization.

## Data Availability

The original contributions presented in the study are included in the article; further inquiries can be directed to the corresponding author.
